# Incomplete Normalization of Regulatory T-Cell Frequency in the Gut Mucosa of Colombian HIV-Infected Patients Receiving Long-Term Antiretroviral Treatment

**DOI:** 10.1371/journal.pone.0071062

**Published:** 2013-08-15

**Authors:** Cesar M. Rueda, Paula A. Velilla, Claire A. Chougnet, Maria T. Rugeles

**Affiliations:** 1 Grupo Inmunovirologia, Universidad de Antioquia, Medellín, Antioquia, Colombia; 2 Division of Cellular and Molecular Immunology, Cincinnati Children's Hospital Research Foundation, Department of Pediatrics, University of Cincinnati College of Medicine, Cincinnati, Ohio, United States of America; University of Hawaii, United States of America

## Abstract

**Introduction:**

To evaluate the effect of late initiation of HAART and poor immune reconstitution on the frequency of regulatory T-cells (Treg) in the peripheral blood and gut of HIV-infected patients, we studied Colombian HIV-infected patients who had been on suppressive HAART for at least one year. They had undetectable viremia but were either immunological responders (HIR); (CD4 counts >500 cells/µl) or non-immunological responders (NIR); (CD4 T-cell count <300 cells/µl). Untreated HIV-infected patients and uninfected controls from the same region were also evaluated.

**Methods:**

Frequency and phenotype of regulatory T-cells (Treg) were analyzed in gut biopsies and blood samples. The functional effect of Treg depletion on CMV and HIV responses was determined. Markers of immune activation and circulating LPS levels were quantified.

**Results:**

Untreated patients exhibited high Treg frequency in PBMC and gut, and their Treg express high levels of CTLA-4 and PD-1. Although HAART significantly decreased mucosal Treg frequency, it did not normalize it in any of the treated groups (HIR and NIR patients). Treg normalization was observed in the blood of HIR patients following HAART, but did not occur in NIR patients. Treg from HIV-infected patients (treated or not) suppressed HIV and hCMV-specific T-cells from gut and blood. Plasma LPS levels and percentage of HLA-DR+CD38+ T-cells were significantly elevated in all infected groups compared to controls.

**Conclusions:**

These findings suggest that control of viral replication is not sufficient to normalize gut Treg frequency in patients, independent of their response to HAART. Furthermore, persistence of functional Treg in the gut appears to be associated with the failure of HAART to repair mucosal damage.

## Introduction

HAART has significantly prolonged the life expectancy of HIV-1-infected patients [1]. In the majority of treated patients, plasma VL becomes undetectable while peripheral blood CD4 counts increase [2]. However, approximately 20% of HAART-treated patients exhibit a discordant phenotype, called “non-immunological response” characterized by undetectable VL but poor recovery of their CD4 counts [3], [4]. Clinical and epidemiological factors associated with this non-immunological response to HAART include duration of infection, presence of co-infections, adherence to therapy and lower CD4^+^ T-cell count at baseline [5].

In many developing regions, HIV-infected patients often initiate HAART when their CD4 counts are 200 cells/ul or less [6], [7], which is much lower than the previous international guidelines (350 cells/ul) [8], [9]. Of note, the new recommendation is to offer HAART to all patients regardless of their CD4 counts [10]. Unfortunately, the Colombian health care system has very limited resources. Consequently, diagnosis of HIV infection occurs at an advanced stage in many Colombian HIV-infected patients. Because low CD4 counts at the time of HAART initiation has been recognized as a critical factor in poor CD4 reconstitution in these low-income settings [7], [11], many of these patients have poor immunological response to HAART. However, little is known on the impact of poor immune reconstitution on CD4+ T-cell subsets, and particularly about its effect on regulatory T-cell (Treg) dynamics.

Treg are a subpopulation of CD4^+^ T-cells, characterized by high constitutive expression of CD25 and FOXP3, and low expression of CD127 [12]. Treg control both innate and adaptive immune responses [13]. Although the role of Treg in HIV pathogenesis is still a matter of debate, these cells could play a dual role. Treg may have a protective effect during the acute infection controlling the number of infected cells and HIV-associated tissue damage [14], [15]. In contrast in chronic HIV-infected donors, Treg might disrupt the development of efficient effector immune responses; in fact, *in vitro* depletion of CD4^+^CD25^+^ T-cells from PBMC results in increased anti-HIV T-cell responses [16]–[18]. Accumulation of Treg in lymphoid tissues such as tonsils, lymph nodes and gut has been reported in untreated chronic progressors [19]–[24]. In patients with both immunological and virological responses to HAART, Treg frequency decreased in both rectal tissues and peripheral blood while CD4^+^ counts, Th17 and polyfunctional HIV-specific T-cell responses all increased in the gut [19], [20], [25], [26].

The aim of our study was to evaluate whether such immune changes also occurred in patients who start HAART later in the course of disease and are at high risk to have poor immune reconstitution. Samples from Colombian patients on suppressive HAART either with immunological or non-immunological response and untreated patients were evaluated. We studied the mucosal barrier dysfunction, as well as changes in the frequency, phenotype and function of gut and blood Treg.

## Results

### Demographic and clinical characteristics of patients

Thirteen healthy controls (HC), 8 patients on HAART with virologic suppression and restoration of CD4 counts (HAART immunological responses, HIR), 9 HAART patients with virologic suppression but impaired restoration (Non-immunological response, NIR) and 16 untreated (UT) patients with active viral replication were enrolled. HIR and NIR patients had been on antiretroviral therapy for a median of 7 years (25th-75th percentiles: 6–10 years) and 4 years (2–13 yrs) respectively. Duration of HAART at the time of analysis was not sigficantly different between HIR and NIR patients (p = 0.38).

HIR patients had similar CD4 counts as controls subjects (median of 938 and 897 cells/µl, respectively; Table 1). In contrast, NIR patients exhibited low CD4 counts despite prolonged viral suppression (median of 288 cells/µl), in the same range as untreated patients (427 cells/µl), and significantly lower than the CD4 counts of HIR patients. High CD8 absolute counts were also observed in both NIR and untreated patients compared to controls (both p<0.05; Table 1). Although CD4^+^ counts at HAART initiation are not known in these patients, individuals in the NIR group exhibited AIDS related symptomatology when they were first tested for HIV antibodies, whereas only 33% of the HIR patients did (Table 1). Time between diagnosis and HAART initiation was similar in HIR and NIR groups (Table 1).

**Table 1:** Clinical characteristics of healthy controls, HAART patients with immunological and non-immunological response and untreated patients.

	Controls (HC) (n = 13)	Immunological Response (HIR) (n = 8)	Non-Immunological response (NIR) (n = 9)	Untreated (UT) (n = 16)	Statistics^#^
**Age (years)?**	48 (34–58)	45 (37–50)	41 (35–46)	30 (27–49)	p>0.05
**% Female/Male**	30/70	12/88	22/85	0/100	p>0.05
**CD4 counts (cells/µl) ?**	897 (654–1156)	938 (545–1190)	288* ^†^ (164–300)	427* ^†^ (272–641)	*p<0.05 Compared to HC ^†^ p<0.05 Compared to HIR
**CD8 counts (cells/** **µl) ?**	458 (344–583)	993 (659–1901)	1143* (879–1391)	1242* (687–1849)	* p<0.05 Compared to HC
**WHO Staging Systems**	N/A	88% B1 12% B2 0% B3	0% B1 67% B2 33% B3	31% B1 56% B2 13% B3	
**Symptomatology at testing°**	N/A	33%	100%	41%	
**Time of HAART initiation after the HIV diagnosis** **(years) ?**	N/A	1 (0–2)	0 (0–0)	N/A	p>0.05
**Time on HAART (years) ?**	N/A	7 (6–10)	4 (2–13)	N/A	p>0.05
**Viral load (copies/ml) ?**	N/A	46 (40–230)	40 (40–90)	32,907^†^ (10,113– 60,520)	^†^p<0.05 Compared to HIR and NIR

**?**Results are expressed as median (25th-75th percentiles). ^#^Groups were compared by the Kruskal-Wallis test and the Dunn's multiple comparison post-test. **°**Data based on questionnaire. The percentage of symptoms at testing was calculated in the group of patients who answered the question. N/A: not applicable.

### Mucosal barrier dysfunction and activation is not restored by HAART despite virologic suppression

**Figure 1:**
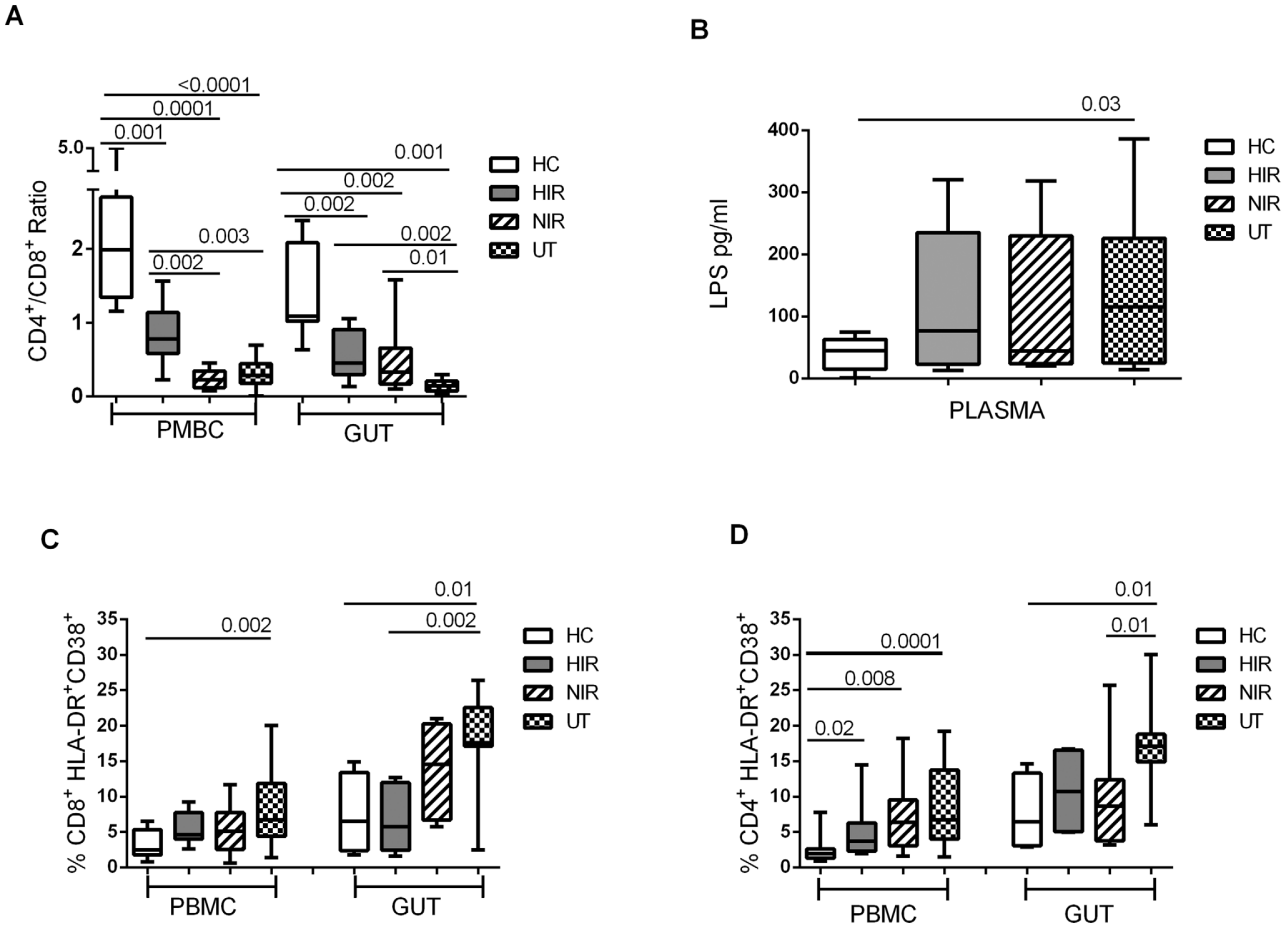
Mucosal barrier dysfunction and activation is not completely restored by HAART. A) Whisker box figures show median and 25–75 percentile of the CD4/CD8 ratio in PBMC and gut from healthy controls (HC), HAART-treated patients with immunological (HIR) and non-immunological response (NIR) and untreated patients (UT). B) LPS concentration in plasma. C) Frequency of CD8 activation (CD8^+^HLA-DR^+^CD38^+^) and D) CD4 activation (CD4^+^HLA-DR^+^CD38^+^) in PBMC and gut. Significant differences are indicated.

The loss of gut integrity is critical to HIV pathogenesis. Increased levels of microbial products in the systemic circulation are associated with a gradual decrease in the number of CD4^+^ T-cells and increased expression of activation markers by multiple types of immune cells [27], [28]. The blood CD4/CD8 ratios were 1.98 and 0.8 in the controls and HIR patients, respectively (p = 0.01; Fig. 1A left panel). This ratio fell to 0.22 in NIR and 0.28 in untreated patients (both p<0.003 compared to controls and HIR patients; Fig. 1A left panel). In the gut, HIR and NIR patients had incomplete reconstitution of CD4^+^ T-cells in the gut after prolonged anti-viral therapy ( p = 0.002; Fig. 1A right panel). Median CD4/CD8 ratio was 0.14 in untreated patients (p<0.002 compared to all the groups; Fig. 1A right panel). Interestingly, NIR patients had greatly decreased blood CD4/CD8 ratios compared to HIR patients (p = 0.002), whereas they had similar gut CD4/CD8 ratios as HIR (p = 0.53).

Untreated patients displayed higher plasma levels of LPS than controls (116 pg/ml vs 45 pg/ml; p = 0.03; Fig. 1B). Circulating LPS levels in HIR and NIR patients were extremely variable and were not significantly different from those of the other groups (Fig. 1B). Untreated patients also exhibited an increased percentage of activated CD8^+^ T-cells and CD4^+^ T-cells (characterized by coexpression of HLA-DR and CD38) in PBMC and in gut (Fig. 1C and Fig. 1D). HAART partially decreased gut and PBMC CD8 and CD4 hyper-activation in NIR and HIR patients (Fig. 1C and 1D).

### High Treg frequency persists in the gut of HAART-treated patients with virologic response

**Figure 2:**
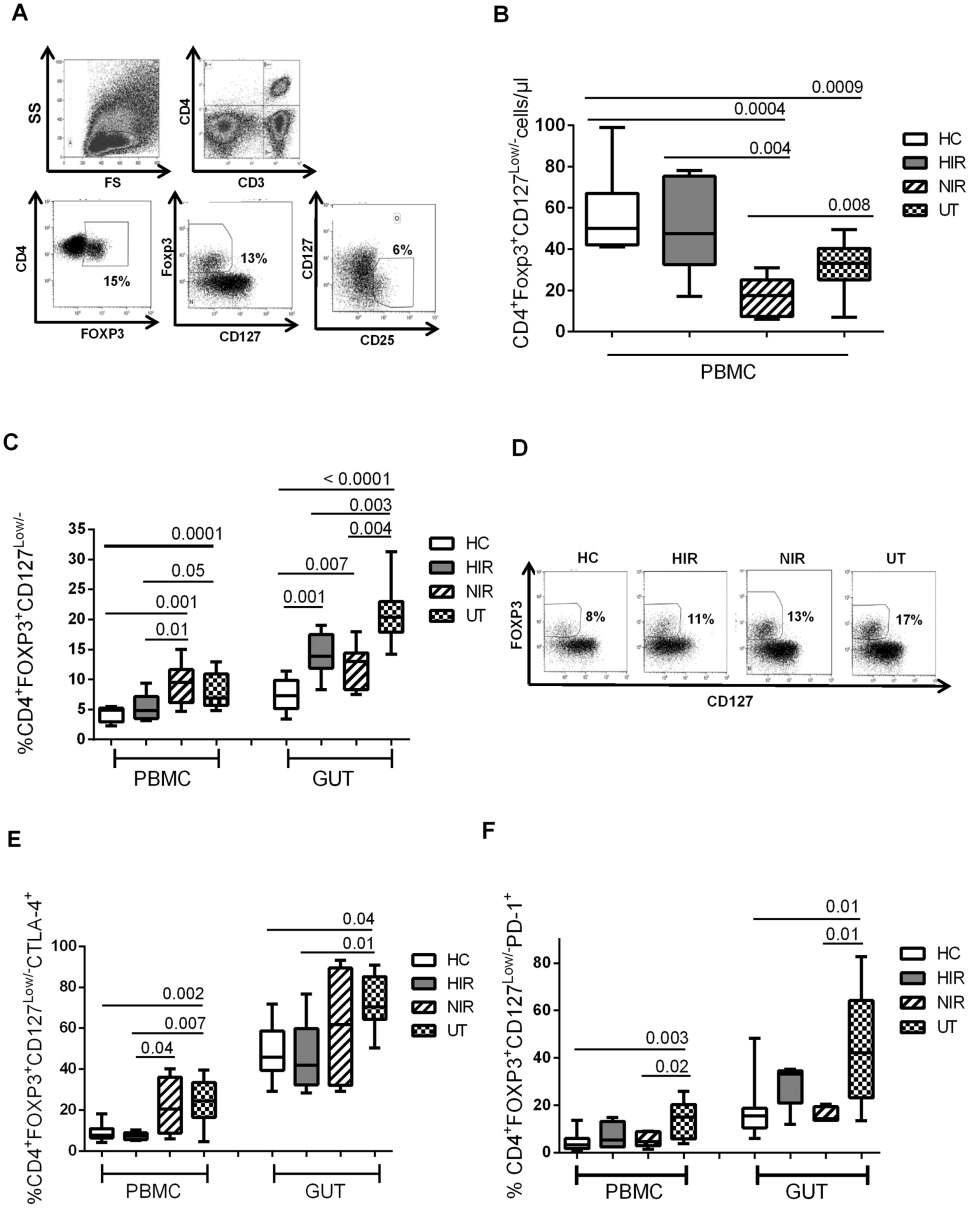
Effect of HAART IN Treg frequency and phenotype in gut and blood tissues. A) Representative figure showing the frequency of Treg in gut in an HIV-infected subject. Three different strategies were used to define Treg in the gated of CD3^+^CD4^+^ T-cells: CD4^+^Foxp3^+^, CD4^+^Foxp3^+^CD127^Low/−^ or CD4^+^CD25^+^CD127^Low/−^. B) Absolute counts of circulating Treg (CD4^+^FOXP3^+^CD127^Low/−^). C) Treg frequency in PBMC and gut. D) Representative examples of Treg (CD4^+^Foxp3^+^CD127^Low/−^) in the different groups of individuals. E) Frequency of Treg expressing CTLA-4^+^. F) Frequency of Treg expressing PD-1^+^ in samples from healthy controls, HIR, NIR and untreated patients.

We next quantified peripheral Treg in the different groups of patients. We analyzed the frequency of Treg using different strategies: CD4^+^FOXP3^+^; CD4^+^FOXP3^+^CD127^Low/−^ and CD4^+^CD25^+^CD127^Low/−^. As shown in Fig. 2A, similar percentage of Treg was detected when they were defined as CD4^+^FOXP3^+^ or CD4^+^FOXP3^+^CD127^Low/−^. In fact, a strong correlation exists between the two measured (r = 0.87, p = 0.001). In contrast, the detection of Treg as CD4^+^CD25^+^CD127^Low/−^ resulted in a lower percentage compared with the other two strategies (Fig. 2A), as previously described [24]. We therefore used CD4^+^FOXP3^+^CD127^Low/−^ as the Treg definition throughout the study. Due to the low absolute CD4 counts in NIR patients, absolute numbers of CD4^+^FOXP3^+^CD127^Low/−^ were decreased in these patients compared to healthy controls (17 vs 50 cells/µl; p<0.0004), HIR (17 vs 47 cells/µl; p<0.004, Fig. 2B) and untreated patients (17 vs 33 cells/µl; p<0.008). Likewise, Treg counts were decreased in untreated patients compared to controls (33 vs 50 cells/µl; p<0.0009). Circulating Treg frequencies were elevated in untreated (7%) and NIR (9%) patients compared to controls (5%) (both p<0.01; Fig. 2C left panel). However, Treg circulating frequencies were similar in HIR patients and controls (Fig. 2C left panel).

In all individuals, Treg proportion was always higher in the gut than in the peripheral blood (1.4-fold higher in controls; 2,2-fold in HIR; 1.4-fold in NIR and 3-fold in untreated patients, all p<0.003, Mann-Whitney tests; Fig. 2C). We thus quantified whether HAART normalized gut Treg frequency and determined whether the effect of viral suppression was different in HIR and NIR patients. As shown in a representative experiment (Figure 2D), the highest Treg frequency was found in untreated patients. Interestingly, Treg frequency remained high in the two groups of HAART-treated patients (HIR and NIR) compared to controls (both p<0.007; Fig. 2C right panel and 2D). Blood Treg frequency was higher in NIR than HIR patients (p = 0.01), whereas gut Treg frequencies was similar (p = 0.423).

Considering all HAART-treated patients, increased gut Treg proportion was positively correlated with CD4^+^ activation (r = 0.47, p = 0.05; data not shown), CD8^+^ activation (r = 0.53, p = 0.03; data not shown) and LPS levels (r = 0.52, p = 0.05; data not shown). In contrast, gut Treg frequencies were negatively correlated with CD4/CD8 ratios (r = −0.51, p = 0.01; data not shown). In untreated patients increased gut Treg proportion was positively correlated with the viral load (r = 0.52, p = 0.01; data not shown).

### Treg CTLA-4+ and PD-1+ are both normalized in HIR patients, but Treg CTLA-4+ remains high in NIR patients

Treg express an arsenal of molecules involved in their suppressive capacity. However, the phenotype of gut Treg has rarely been examined, particularly during HIV infection. We thus compared the phenotype of Treg in the blood and gut compartment in the different groups of HIV-infected patients. In all individuals, the frequency of Treg expressing CTLA-4 and PD-1 was always higher in the gut than in the peripheral blood (Fig. 2E and 2F). The percentage of circulating and gut Treg expressing CTLA-4 was significantly increased in untreated patients compared with controls and HIR patients (Fig. 2E). Frequency of circulating CTLA-4+ Treg in NIR was higher than HIR patients (p = 0.041) but not in gut Treg (p = 0.428) (Fig. 2E). Similarly, PD-1 expression by Treg increased in untreated patients compared to that in controls and NIR patients (Fig. 2F). PD-1 expression by Treg was not significantly different in NIR, HIR patients and controls (Fig. 2F).

### Mucosal Treg of HIV-infected individuals suppress cytokine production and degranulation of effector T-cells

**Figure 3:**
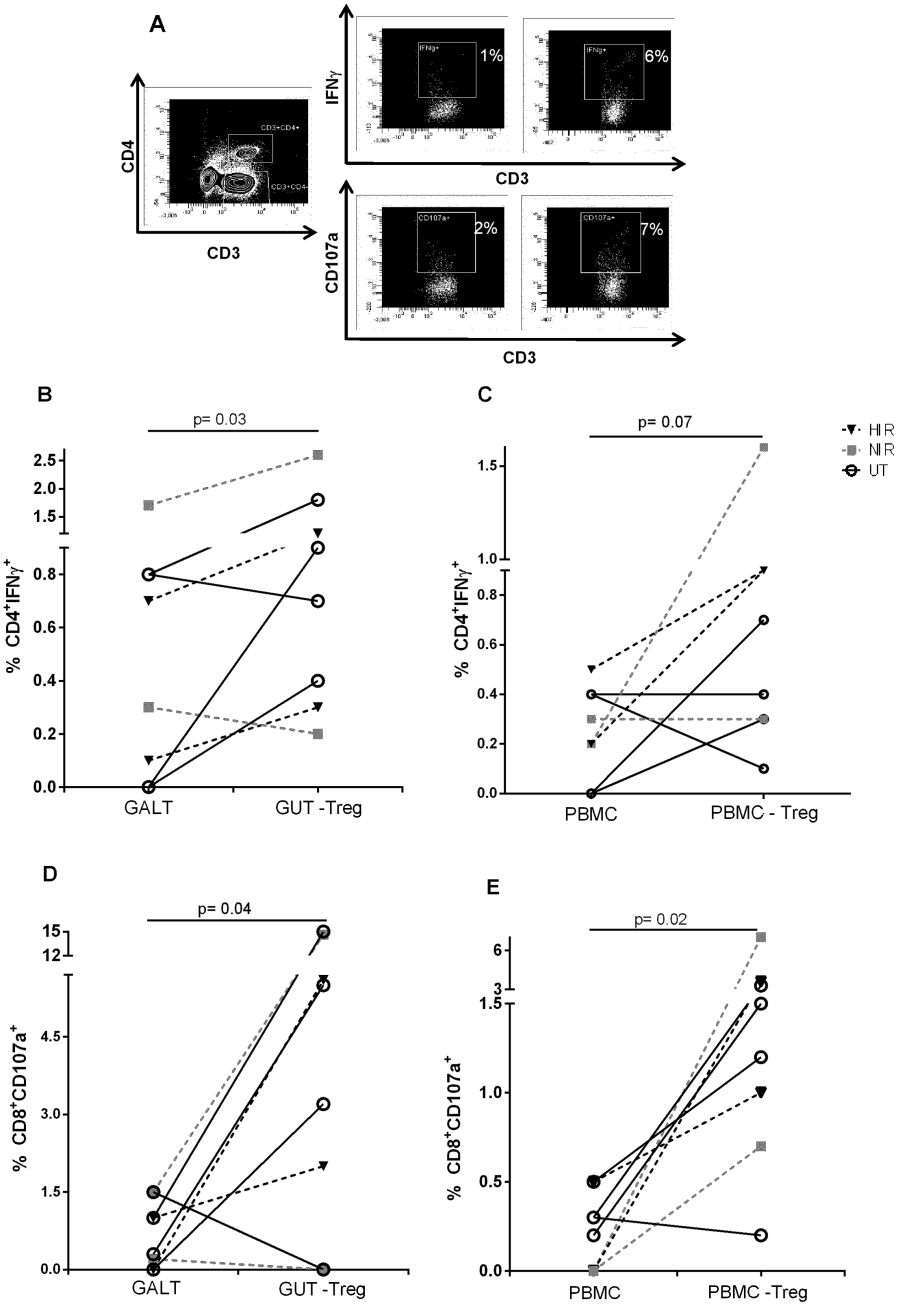
Treg from HIV-infected individuals suppress anti-hCMV immune response. Total rectal cells or Treg depleted were stimulated with 10 μg/mL hCMV pp65-peptide pool in presence of anti-CD28/ CD49d. A) Representative example of intracellular IFN-γ by CD4^+^ T-cells(top) and CD107a expression by CD8^+^ T-cells (bottom). B) and C) IFN-γ production by CD4^+^ T-cells from gut and PBMC, respectively. D) and E) CD107a expression in CD8^+^ T-cells on gut and PBMC, respectively.

We then compared suppression by Treg from HAART (2 HIR and 2 NIR patients) and 4 untreated patients. Depletion of CD25^+^CD127^Low/−^ Treg from gut and PBMC led to an increase in the frequency of hCMV-responsive CD4^+^IFN-γ^+^ in most patients (p = 0.03 and p = 0.07, respectively; Fig. 3A and 3B). Likewise, depletion of Treg increased CD8 degranulation by more than 4-fold (p = 0.04 and p = 0.02 for the gut and PBMC respectively; Fig. 3C and 3D). Interestingly, the suppressive ability of Treg was similar between untreated and HAART-treated patients and between samples from PMBC and gut.

**Figure 4:**
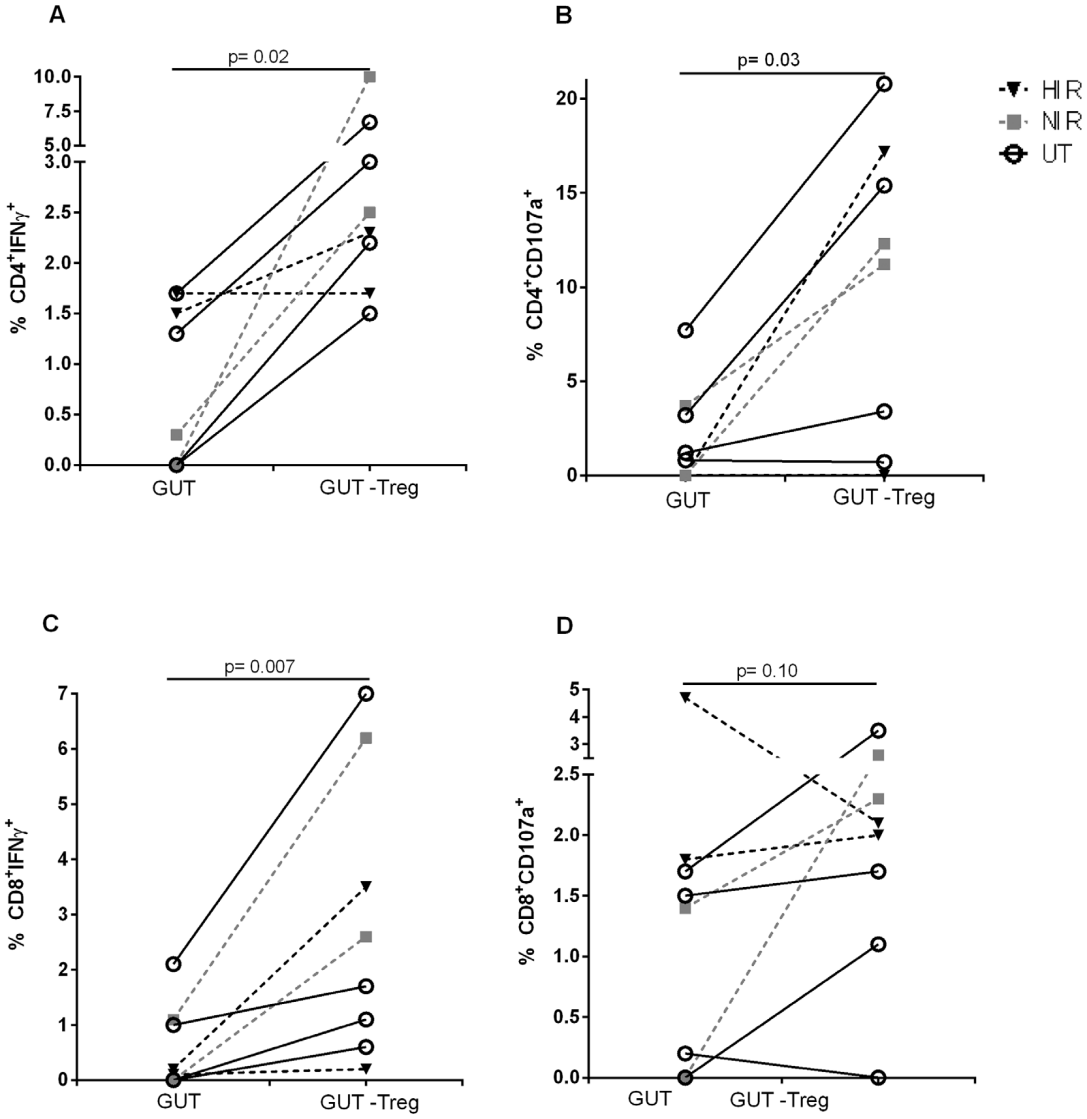
Treg of HIV-infected individuals suppress anti-HIV immune response. Total rectal cells or Treg depleted were stimulated with 10 μg/mL HIV-1 Gag-peptide pool in presence of anti-CD28/CD49d. IFN-γ and degranulation was measured by flow cytometry after 18 h. A) and C) Graphs show mean intracellular IFN-γ expression by CD4^+^ and CD8^+^ T-cells respectively. B) and D) Expression of CD107a by CD4^+^ andCD8^+^ T-cells respectively.

Similarly, specific HIV-Gag responses were inhibited by Treg. Indeed, after Treg depletion, IFN-γ production by gut CD4^+^ or CD8^+^ increased by ∼6-fold (p = 0.02) and ∼14-fold (p = 0.007), respectively (Fig. 4A and 4C). Degranulation of gut CD4^+^ T-cells was also dampened by Treg, as their depletion led to a >10-fold increase in the expression of CD107a by non-Treg (p = 0.03; Fig. 4B). Similar results were seen in treated and untreated patients. CD8 degranulation in response to HIV-peptides was not strongly increased by Treg depletion (p = 0.1; Fig 4D).

## Discussion

HIV replicates in lymphoid organs, mainly in GALT, where most of the body CD4^+^ T-cells reside. In this tissue, HIV induces severe structural and functional damage, leading to translocation of commensal bacteria or other pathogenic microorganisms from the lumen to the systemic circulation [27]. Subsequent activation of the immune system has been implicated as a mechanism underlying exacerbated activated-induced cell death [28]. The untreated patients that we studied had severe decreases in CD4/CD8 ratios, increased circulating levels of LPS and increased immune activation. These defects were not fully normalized by HAART, which is in agreement with previous studies [23], [29], [30]. In our patients, the percentage of gut HLA-DR^+^CD38^+^ T-cells and the plasma LPS levels were negatively correlated with the gut CD4/CD8 ratios, suggesting a persistent vicious cycle, in which sustained microbial translocation and limited immune reconstitution exacerbate each other, leading to persistent dysfunction of the mucosal barrier [31].

We found high Treg frequency in the gut of untreated patients compared with controls and HAART treated patients, a finding consistent with previous studies [19], [20], [32]. Importantly, both NIR and HIR patients exhibited persistent high Treg frequency in the gut despite HAART. Together with the fact that HAART did not normalize plasma LPS levels and immune activation, these data suggest two non-exclusive mechanisms, namely low-grade HIV replication and persisting mucosal damage. Both are implicated in continued inflammation as well as in Treg accumulation [30]. Corroborating our hypothesis, a positive correlation was found between the gut Treg frequency and T-cell activation or LPS plasma levels. Our data are in contrast with two previous studies showing that HAART normalizes Treg frequency in the gut [19], [20]. Notably, these two studies were conducted in the USA and Germany, whereas our study is the first to examine gut Treg frequency in patients living in a low-income country. The study populations are thus very different: HAART initiation occurred later in our population, and the prevalence of intestinal pathogens is likely higher, although it was not measured in our study. Both factors are likely to contribute to persistent mucosal damage and inflammation. In addition, the relationship between mucosal damage and Treg is likely bi-directional. Previous reports have indeed shown that increased Treg frequency is associated with extensive fibrosis in gut and lymph nodes, including in HAART-treated patients, due to transforming growth factor beta 1(TGF-β)-mediated collagen deposition [33]–[36].

We also studied the effect of HAART on Treg frequency in the blood, and found that HAART normalized circulating Treg in HIR patients but not in NIR patients. One possible explanation for these data is that NIR patients still exhibit a pattern of generalized hyper-activation as evidenced by persisting increased levels of activation markers by circulating T-cells, whereas immune activation was by and large controlled in HIR patients (Fig. 1D). These data are consistent with previous studies of HIR [26] and NIR patients [37], and confirm that, in addition to overall poor CD4 reconstitution, NIR patients exhibit persistent qualitative defects in CD4+ T-cell subsets. This could contribute to the fact that polyfunctional T-cells are less frequent in NIR than in HIR patients [38].

Cytokine production of gut T-cells in response to Gag peptides was augmented after Treg depletion, suggesting that Treg dampen anti-viral immune responses *in vivo,* which is consistent with previous studies exploring gut Treg functionality in HIV-infected patients [16]–[18]. Gut CD8^+^ and CD4^+^ T-cells were able to degranulate (as indicated by CD107a expression) in response to Gag peptides, as already reported for circulating and gut T-cells [39], [40]. Treg generally controlled this function in CD4+ T-cells, as evidenced by the significant increase in CD107a expression after Treg depletion, but their effect on CD8 degranulation was inconsistent. However, it must be noted that Treg were able to control IFN-γ production by HIV-specific CD8+ T-cells. Together, these data suggest that Treg are globally functional, although the magnitude of their effect is not consistent across all cellular functions. Such differential suppression had already been reported by Elahi et al, as Treg had a more marked effect on CD8 cytokine production than on proliferation [41]. Because upregulation of CTLA-4 and PD-1 are associated with increased suppressive capacity of Treg [42], we also characterized the expression of these molecules by Treg. Gut Treg from untreated patients exhibited increased expression of CTLA-4 and PD-1 compared to those from treated patients, consistent with previous reports [21], [22], [32]. However, they were not more suppressive *in vitro*. One possible explanation for this result is that these molecules mainly control dendritic cell activation, which was not tested in our *in vitro* assay using peptides as stimulator [43], [44].

In conclusion, our data suggest that the effect of HAART on CD4 subsets is complex. HAART did not normalize mucosal Treg in patients from Colombia, who are representative of HAART-treated patients in low-income countries. Partial normalization was observed in the blood, but was limited to the subsets of patients with overall CD4 reconstitution. It will thus be important in the future to clearly identify the predictors of poor Treg normalization in our region and other similar countries. These data also provide further evidence that patients eligible for HAART should be identified early in the course of disease.

## Materials and Methods

### Subjects and sample collection

A cross-sectional study was performed in 2008–2010 enrolling subjects from Medellin (Colombia). Four groups of subjects were included in the study: (i) HIR patiens: HIV-infected individuals treated with HAART for at least one year, defined as patients with both a virological response (undetectable viral load <40 HIV RNA copies/ml) and CD4 reconstitution (CD4 counts of >500 cells/µl); (ii) NIR patients: HIV-infected individuals treated with HAART for at least one year, defined as patients with maintained viral suppression (undetectable viral load) but low levels of CD4 reconstitution (CD4 T-cell count of <300 cells/µl); (iii) untreated HIV-infected patients (some of them recently diagnosed) with active viral replication (>10,000 HIV RNA copies/ml); and (iv) healthy HIV-uninfected controls. Patients were excluded for non-adherence to HAART, acute illness and opportunistic infections at the time of sampling. HAART patients with viral blips (>50 but <300 copies/ml) were included. The percentage of patients with classical HAART regimen (three drugs: 2 NRTIs + a PI/NNRTI/II) was 86% in HIR and 89% in NIR.

The demographic and clinical characteristics of the enrrolled individuals are shown in Table 1. The absolute T-cell number was determined in venous blood samples and plasma HIV RNA levels were assessed by RT-PCRq (COBAS AmpliPrep/TaqMan). All individuals answered a questionnaire and signed an informed consent prepared according to the Colombian Government Legislation (Resolution 008430-1993). The study was approved by the Bioethical Board for Human Research from University of Antioquia.

### Isolation of cells from peripheral blood and gut

PBMC were obtained from 10 ml of heparin venous blood samples by centrifugation on Histopaque (Sigma-Aldrich, St Louis, MO, USA) for 30 min at 400 g. Biopsy tissue was obtained by rectosigmoidoscopy in the rectum at 10 cm from anal verge. A flexible sigmoidoscope with single endoscopy Biopsy forcep FB-24K-1 (Olympus America Corp, Melville, NY, USA) was used. At each procedure, 20 tissue samples were obtained. Samples were processed using 0.5 mg/ml collagenase type II from Clostridium histolyticum (Sigma) diluted in RPMI 1640 and 7.5% FCS plus 100 U/ml penicillin and 100 ug/ml streptomycin (Gibco-BRL; Grand Island, NY, USA), for 30 min at 37°C with shaking. After collagenase digestion, biopsy fragments were further disrupted by repeated passage through a 30 ml syringe with a blunt end 16 gauge needle (Stem Cell Technologies, Vancouver, BC). Rectal cells (RC) were isolated from the fragments by passage through a 70 μM nylon strainer (Falcon, Lincoln Park, NJ, USA). PBMC and RC were washed with Dulbeco's PBS (DPBS) (Sigma-Aldrich) to remove excess of histopaque and collagenase. Subjects with nodular lymphoid hyperplasia, ulcers, diverticulum, adenoma and other benign or malign neoplasias were excluded from the study. The macroscopic evaluation of rectosigmoidoscopies was normal in all individuals.

### Phenotypic characterization by flow cytometry

For T-cell counts, 150 µl of whole blood was incubated for 30 min at 4°C with anti-CD3-FITC (clone UCHT1), anti-CD4-PE-Cy5 (RPA-T4), and anti-CD8-PE (RPA-T8) (BD Pharmingen, San Diego, CA, USA); red blood cells were eliminated by lysis buffer (BD Biosciences) for 10 min; cells were washed twice with DPBS before analysis by flow cytometry.

PBMC and RC (1×10^6^ cells) were distributed into cytometry flow tubes and treated with 20 ug/ml of human IgG to block Fc receptors for 15 min, then surface-stained for 30 min at 4°C with anti-CD4-ECD (SFCI12T4D11) from Beckman Coulter (Fullerton, CA, USA), anti-CD3-PE-CY7 (UCHT1), anti-CD25-PE-CY5 (IL-2-Rα, clone BC96), anti-CD127-PE (IL-7-Rα, clone RDR5), anti-HLA-DR-PE (LN3), anti-CD38-FITC (HIT2), and anti-Programmed Death-1 (PD-1)-PE (CD279, MIH4), from (e-Bioscience, San Diego, CA, USA). Cells were washed with DPBS, fixed/permeabilized with FOXP3 staining buffer set (e-Bioscience), and stained intracellularly using anti-FOXP3-FITC (PCH101, e-Bioscience) and anti-CTLA-4-PE (CD152, BNI3, BD Pharmingen) for 30 min at 4°C. At least 1×10^5^ events were acquired using the flow cytometer FC500 (Beckman Coulter) and analyzed using Kaluza Software (Beckman Coulter). Appropriate isotype-matched control antibodies were included to define positive thresholds of HLA-DR and CD38 expression on the CD3+CD4+ or CD3+CD8+ gates. The strategy gate for Treg was defined in a negative biological population (CD3−CD4−), as previously described [45].

### Functional assays

PBMC or RC were stained with anti-CD25-PE-CY5 and anti-CD127-PE; the resting Treg cells were isolated to purity above 90% by CD25+CD127^Low/−^ sorting (MofloTM XDP, Beckman Coulter). Freshly isolated PBMC or RC and cultures depleted of Treg were cultured in 48-well plates at a concentration of 4×10^5^ cells per well in 400 μL of RPMI 1640 medium (Invitrogen, OR, USA) with 10% FCS. The cells were incubated in the presence of 10 μg/mL brefeldin A (Sigma-Aldrich), 2 uM monensin, 1 µg/mL anti-CD28 (CD28.2), 1 µg/mL anti-CD49d (9F10) (eBioscience) and 5 µL CD107a-APC (H4A3) (BD Pharmingen), and stimulated for 18h with 10 μg/mL of the hCMV pp65-peptide pool (138 peptides) or the HIV-1 Consensus B Gag-peptide pool (138 peptides). Each pool was 15aa in length, with 11aa overlaps between sequential peptides. Cells cultured with anti-CD28 and anti-CD49d served as background.

Cellular activation and intracellular cytokines were measured, using anti-CD3-FITC (UCHT1), anti-IFNγ-PE-CY7 (clone B27, BD Pharmingen), and anti-CD4-PB (OKT4, e-Bioscience). The samples were acquired on the FACS CANTO II (BD Pharmingen). Analysis was performed using FACSDiva software (BD Pharmingen) and results were expressed as a percentage of responding T-cells minus the expression in unstimulated cells.

### Plasma LPS levels

Plasma samples were inactivated by heating at 70°C for 10 min. Duplicates of 50 µl of samples and standard endotoxin solutions were added in 96-well flat-bottom plates (Costar-Corning, Lowell, MA, USA), and then incubated with Limulus Amoebocyte Lysate (LAL) for 10 min and substrate solution for 6 min at 37°C. The reaction was stopped with acetic acid (25% v/v glacial acetic acid in water). The assay was performed according to manufacturer's instructions by the endpoint chromogenic LAL assay QCL-1000 (Lonza Inc, Allendale, NJ, USA) (sensitivity range 0.1–1.0 EU/ml). The background attributable to the turbidity of the diluted plasma was subtracted.

### Statistical Analysis

Statistical analyses were carried-out using GraphPad Prism 5 (San Diego, CA, USA). Comparisons of medians among groups were performed by Kruskal-Wallis test and Dunn's post-test. Both groups of HIV infected patients were compared by U Mann Whitney or Wilcoxon test depending on the analysis. Correlations were made with Spearman test. In all analyses and p values less than 0.05 were considered to be significant.
